# Designing effective exercise intervention trials for prostate cancer cohorts: a qualitative study on experiences and views of exercise oncology researchers

**DOI:** 10.1186/s13102-023-00756-7

**Published:** 2023-10-30

**Authors:** Kira Murphy, Suzanne Denieffe, Bróna Kehoe, Dayle Hacking, Ciaran M. Fairman, Michael Harrison

**Affiliations:** 1 Department of Sport and Exercise Science, South East Technological University, Waterford, Ireland; 2 UPMC Hillman Cancer Center, Whitfield Hospital, Waterford, Ireland; 3 School of Humanities, South East Technological University, College Street Campus, Waterford, Ireland; 4 Department of Exercise Science, Arnold School of Public Health, University of South Carolina, Columbia, SC USA

**Keywords:** Prostate cancer, Exercise oncology, Expert opinion, Trial design

## Abstract

**Background:**

Exercise intervention research has shown promising results in preventing and reversing the side effects caused by prostate cancer and its’ treatment. However, there are still unanswered questions and the need for additional research. As the field of exercise oncology in the context of prostate cancer presents unique challenges and complexities, seeking the advice of experienced exercise oncology researchers before initiating a similar trial could help to design more effective and efficient studies and help avoid pitfalls.

**Methods:**

A qualitative descriptive study design and a nonprobability, purposive sampling method was employed. An interview guide was developed and included topics such as recruitment, retention, programme goals, research design, health considerations, treatment considerations, adverse events, exercise prescription and outcome tools. Individual semi-structured interviews were conducted and interviews were transcribed and analysed using thematic analysis.

**Results:**

Eight individuals with extensive experience working with prostate cancer patients in exercise oncology research settings were interviewed. Four main themes and seven subthemes were generated and supported by the data. Theme 1 highlighted the critical role of recruitment, with associated subthemes on recruitment barriers and recruitment methods. Theme 2 explored the positives and negatives of home-based programmes. Theme 3 focused on specific health characteristics, exercise prescription and outcome measure factors that must be considered when working with prostate cancer cohorts. Finally, theme 4 centered around the emotional dimensions present in exercise oncology trials, relating to both researchers and study participants.

**Conclusion:**

Exercise oncology remains a challenging area in which to conduct research. Learning from experienced personnel in the field offers valuable information and guidance that could impact the success of future trials.

**Supplementary Information:**

The online version contains supplementary material available at 10.1186/s13102-023-00756-7.

## Introduction

An inability to establish efficacy has been stated as the primary source of failure in clinical trials [1]. This may be caused by flawed trial design, inappropriate endpoints, or inadequate sample size due to issues with recruitment and retention [2]. The effective design of trials is becoming much more critical, particularly for complex interventions. The Medical Research Council framework emphasise the importance of consultations with stakeholders and the conduct of feasibility and pilot trials [3]. Combining the expertise of multiple individuals has also been shown to improve decision making and aid in the evaluation process of a design [4]. Consulting experienced personnel in the relevant field prior to commencing a trial can aid in the identification of potential pitfalls and effective strategies. This could potentially lead to more effective, efficient, and impactful studies in the future.

Prostate cancer is the second most common cancer in men worldwide [5, 6]. There is a growing body of literature that has been systematically reviewed, investigating the benefits of exercise to men with prostate cancer [7–12]. These reviews have reinforced the benefits of exercise among this cohort of patients but have also highlighted the significant heterogeneity among studies, including variations in study size, exercise prescription, outcome measures, measurement tools and improvements in participants’ health and quality of life [7–12]. Prostate cancer patients and survivors have unique treatments, side effects, exercise experiences and barriers that must be considered during the research planning process.

To date, qualitative research regarding cancer rehabilitation programmes has mainly focused on the experiences, attitudes, and knowledge of cancer patients, cancer survivors, and healthcare professionals [13–16]. To the best of our knowledge, no research has been carried out specifically on exercise oncology researchers. This qualitative study therefore aims to explore the experiences and views of the prostate exercise oncology research community with involvement in intervention supervision and research design. Their experiences and views have the potential to optimise future exercise interventions in the research setting and to improve evaluation trial design.

## Method

### Study design

A qualitative descriptive study design was used for this research. This study is reported according to the Standards for Reporting Qualitative Research (SRQR) [17].

### Participants

The population of interest were individuals who had worked directly with prostate cancer patients in a research setting involving an exercise intervention. Individuals within the same research group was permissible if they had fulfilled distinctly different roles within the research team. Authors of this study were excluded from participation.

The interviewees provided written informed consent and confirmed consent verbally prior to the commencement of the interview. Ethical approval was obtained from Waterford Institute of Technology (WIT) Research Ethics Committee. This study was performed in accordance with the Declaration of Helsinki.

### Sampling method

A purposive expert sampling method was used for this study, whereby the research team targeted published experts in the field due to their unique qualities and experiences [18]. Invitation emails containing a study information sheet were sent to potential interviewees. The information sheet outlined the study’s aims, topics for discussion during the interview and approximate time commitment. The sample size was determined based on theoretical saturation [19]. This was achieved by conducting data review and analysis in conjunction with data collection until saturation was reached [20].

### Procedure

Individual semi-structured interviews based on an interview guide were used to collect data. This interview guide was developed specifically for this trial (Additional file 1). This approach was used as it offered guidance as well as flexibility [21]. Each interview was undertaken online by KM, who has extensive clinical experience working with prostate cancer patients but not in an exercise oncology setting. Interviews were audibly recorded with permission from the interviewees. Interviewees were offered the opportunity to view the transcript of their interview on completion.

The research team held a pilot interview prior to data collection which enabled the interview protocol to be tested, highlighting possible practical issues or difficulties prior to recruitment [22]. No changes were made after the pilot interview.

### Data analysis

Data was analysed using the reflective thematic approach as developed by Braun and Clarke [23, 24]. Initial data analysis was carried out by KM and discussed in depth with all other authors as the analysis proceeded from codes to subthemes.

Phase 1 encompassed data familiarisation, comprising of iterative listening of the complete dataset, transcription and identification of potential initial patterns. In phase 2, transcripts were imported into NVivo 12 (QSR International, Doncaster, UK) and initial codes were created by actively analysing each line. In phase 3, the research team engaged in discussions to interpret the relationships between associated codes, resulting in the emergence of subthemes and overarching main themes across the entire dataset. This led to the development of an initial thematic map. Phase 4 involved refining the themes at two levels: analysing and revising the coded extracts for coherence and verifying the accuracy of the themes in representing the entire dataset in relation to the research question. In phase 5, main themes were given appropriate and defined titles to capture the audience’s attention while accurately conveying the study’s overall message. Finally, in phase 6 a comprehensive report was compiled, and a final inspection was completed.

### Role of funding source

The funder played no role in the design, conduct, or reporting of this study.

## Results

Eight individuals from 6 different research teams were interviewed over a two-month period in 2021. Ten initial invites were sent to recognized experts and primary authors of peer-reviewed papers in the field of prostate cancer and exercise oncology. Three individuals accepted, four individuals declined or did not respond, and three individuals suggested other members of their research team as it was felt they could offer more valuable insights into the research question. This resulted in an additional five individuals being invited, all of whom accepted. All interviewees had experience working on funded published research studies. Many had experience working on multiple trials, ranging from feasibility trials to randomised control trials. Interviewee characteristics are shown in Table 1.

**Table 1:** Interviewees Characteristics

Characteristic		%
Sex, %(n)	*Male*	**50% (4)**
	*Female*	**50% (4)**
Education, % (participant identifier)	*Undergrad*	**25% (participant 2,5)**
	*Masters*	**25% (participant 6,8)**
	*PhD*	**50% (participant 1,3,4,7)**
Location of research, *%(n)*	*West America*	**25.0% (2)**
	*South America*	**12.5% (1)**
	*Ireland/United Kingdom*	**50% (4)**
	*Europe (other)*	**12.5% (1)**
Professional expertise, % (participant identifier)	Design and study Implementation	**50% (participant 1,3,4,7)**
	Study implementation	**50% (participant 2,5,6,8)**
Years of experience in exercise oncology (approx.), % (participant identifier)	1-5 years	**37.5% (participant 2,3,5)**
	6-9 years	**12.5% (participant 6)**
	10+ years	**50% (participant 1,4,7,8)**

The mean duration of the eight interviews was 67 min ± 15 min. None of the interviewees requested to review their respective transcripts. Four main themes and seven subthemes relating to recruitment, homebased programmes, prostate cancer-specific considerations and emotional dimensions were generated and supported by the data.

### Theme one: Recruitment is a critical challenge that must be anticipated and prepared for

The significance of trial recruitment was a topic of discussion amongst all interviewees and was identified as a major challenge within the realm of exercise oncology research. Many played an active role in recruitment such as attending hospital clinics, sending out invites or being the main contact point after an oncologist deemed a patient eligible and willing to take part in a trial. Within this theme, two distinct subthemes emerged: Recruitment barriers and recruitment methods.

### Recruitment barriers

Interviewees identified three main reasons as to why patients declined to participate in exercise trials: lack of interest, travel burden and time commitment. Issues around time commitment were reported to be more apparent among the metastatic prostate cancer population.

*“And they just think…like the disease had spread to my bones, my oncologist told me I’ve got three years to live. Well, the last thing I want to be doing is spending my time exercising even though it may help” P3*.

Recruiting as the patient is beginning active treatment was also seen as more complex, and interviewees warned researchers targeting this timepoint to be prepared for additional obstacles.

*“You know there are just a lot more obstacles to recruiting someone when they’re actively in treatment and not only trying to coordinate with time, their feelings and how they’re fatigued or whatever and trying to overcome that.” P6*.

Clinical workload, limited appointment times and virtual consultations were also highlighted as barriers when recruiting through hospital clinics.

### Recruitment methods

Three main recruitment methods were utilised among the researchers interviewed: direct recruitment from hospital clinics, open invites, and state registry invites.

All interviewees had experience recruiting directly from hospital clinics and agreed that collaboration with the clinical team was critical. It was indicated that having a referrer or healthcare personnel on the research team can be beneficial to invoke a sense of responsibility for the trial’s success within the hospital. Involving them in the developmental stages of the trial before grant application was suggested to reinforce a sense of ownership. Being a physical presence in the hospital, attending multi-disciplinary team meetings, organising weekly calls or meetings with clinical leads, and overall building a rapport with the clinical team was seen as critical by the interviewees if recruiting from a hospital clinic.

Open invites sent through cancer support organisations were also deployed by some. However, these open calls created huge burden of follow-up as pre-screening was not possible. This resulted in many patients not meeting eligibility criteria, so the approach was not carried forward to future trials.

*“It creates a lot of follow up, so you’re on the phone constantly for weeks and weeks, and you end up with, I don’t know a handful (of participants)” P1*.

Recruitment through the state cancer registry was considered a more viable option compared to open invites as pre-screening for specific parameters was possible. In some cases, it had become the primary method of recruitment.

*“From clinic to our next strategy which I am a fan of…we are kind of lucky to have access to the state registry…its rather time-consuming but we have been more successful with that” P1*.

### Theme Two: There are both positives and negatives to home-based programmes

Several interviewees had experience with home-based exercise programmes, with some having to transition their supervised trials to the home setting during the Covid-19 pandemic. Home programmes were reported to tackle recruitment barriers such as travel and time commitment and were particularly popular with younger patients that may be juggling work and family commitments.

*“Having the accessibility, just doing it in their home. I think it’s actually helped our recruitment.” P8*.

However, in terms of overall benefit, most interviewees still reported supervised, in person, exercise classes to be superior in terms of training benefits.

*“Well, I guess, with our home programme, I think some preliminary data for us seems like they’re not getting the same like training benefit that those in person are” P6*.

A possible lack of personal motivation, lack of equipment, difficulty achieving adequate exercise intensity, reduced ability to coach, loss of peer and social support and a greater need to trust patients were cited as some of the disadvantages experienced with home programmes.

*“In terms of it can take more motivation on the other individual’s part, to show up and be accountable to themselves…I think we find people are willing to show up when they are accountable to others and a group.” P6*.

A blended approach that combines supervised and home-based programmes with the option for participants to fully transition to a home programme was suggested by some interviewees to be a feasible strategy. However, it was highlighted that accurately monitoring activity and intensity in the home environment can present challenges, particularly when adherence to a specific exercise prescription is required for research purposes.

*“We have them using, you know, wearable technology, to try, so to try to verify what they’re doing. For resistance, I’ve got no way of verifying it entirely, you know, you told me you did this, I’ve got to take your word for it”. P4*.

### Theme Three: There are specific health characteristics, exercise prescription and outcome measure factors that must be considered when working with prostate cancer cohorts

All interviewees gave their views on designing and conducting exercise trials for this cohort of patients based on their direct experience.

### Health characteristics

Interviewees reported that prostate cancer and its treatment side effects posed challenges for patients, potentially affecting their ability or willingness to participate in exercise. The most common side effects observed in the non-metastatic prostate cancer population were incontinence and fatigue. Given the potential changes in physical and mental health among this cohort over the duration of a study, a flexible exercise programme was stated to be hugely valuable and likely necessary.

*“If you have a step-by-step protocol you know, can it be varied, can you come off script a little if they are not hitting or increasing by a certain point” P5*.

Interviewees with experience working with the metastatic prostate cancer population, stated that bone pain due to bony metastases and the safety issues around loading effected regions was problematic and led to restricted exercise prescriptions. Disease progression and active therapy such as chemotherapy and radiation therapy were noted as more common in this cohort of patients and must be considered. Interviewees found patients generally managed to continue with the exercise intervention with some adjustments during active treatment if they were already established on the exercise programme.

*“Actually, they were still motivated to take part…chemotherapy isn’t necessarily the excluder that we thought it might be or didn’t hinder our patients, and they carried on.” P2*.

One of the most common issues that caused the interviewees to alter their planned exercise prescription was not cancer related but was associated with existing comorbidities or lack of mobility.

*“I mean, it really depends on what other conditions they come along with, really, is one of the biggest things you know…Things like arthritic joints or particularly painful joints and that can make it challenging.” P5*.

Some interviewees found a “introductory phase” at the beginning of their study, where the main focus was on technique, body awareness, and flexibility to be beneficial.

*“But we found it to be really helpful to spend that time on mobility exercises to get more range of motion through their hips and back and then just teaching them body awareness…and then they’re able to progress quicker.” P8*.

### Exercise prescription considerations

When asked about the exercise programme for this cohort, generally, the advice was to start and progress slowly.

*“And if they haven’t ever exercised before, you don’t want to suddenly give them a huge amount of load, and they kind of can’t walk for a week because they’ve got huge amount of thigh or muscle DOMS* (delayed onset muscle soreness)*”P5*.

Progression and programme intensity were suggested to be based on the previous session as some participants will progress slower than predicted. It was felt that prostate men have a remarkable ability to adapt to exercise and can be pushed harder than other cancer patients as they move through the programme.

*“I think they have a great capacity to respond, I think they’re just older men who have really, really low testosterone as opposed to kind of low.” P4*.

High intensity was suggested by some, particularly with resistance training, to aid adaptation when trying to reduce session time or volume. However, those with experience delivering a high-intensity programme, spoke of the challenges that came with higher intensities and suggested to err on the side of caution, remembering these patients may be cardiovascularly compromised.

*“It required one to one supervision….it required a high degree of motivation by those men, is it possible? The answer is: it is possible. Is it practical? I don’t think so.” P4*.

### Outcome measures

Interviewees expressed their views on the most appropriate fitness outcome measures for this cohort of patients in a research setting. The two main fitness elements discussed were cardiovascular fitness and strength.

Regarding cardiovascular fitness, some interviewees emphasised cardiopulmonary exercise testing (CPET) as the gold standard, suggesting limitations with field tests such as a ceiling effect. However, the majority regarded field tests to be appropriate and reduced equipment and time resources. Some interviewees argued that field tests were more accessible and could allow researchers to bring their programme out into the community and home setting.

With regards to strength tests, one-repetition maximum was deemed appropriate by all with strength test experience. Field tests such as the 30s sit to stand were used as a substitute when programmes were tested out in the community or the home setting.

Quality of Life (QOL) questionnaires were reported as one of the most tedious and irritating outcome measures for participants and researchers. Methods used to administer questionnaires varied between electronic and hardcopy, but there was general agreement among interviewees that technology was not a barrier with this cohort. Interviewees stated the completion of QOL questionnaires was high, however the more sensitive sections on sexual health were the areas most likely to be omitted. Interviewees also highlighted that psychological outcomes were as important as physiological outcomes and must be considered when evaluating the success of an exercise intervention.

*“If you design an intervention and nothing changes physiologically, but they’re telling you that their quality of life or their fatigue scores are better, you know that is still a win, and maybe that’s the most important win we have because it’s their life.” P4.*

Another point to note was a perceived lack of control over the standardisation of procedures when outsourcing services, such as Dual Energy X-ray Absorptiometry (DEXA).

### Theme Four: There are emotional dimensions to exercise oncology trials

The emotional dimensions discussed by the interviewees applies to both researchers and the study participants themselves, leading to two distinct subthemes for each group.

### Researchers

The interviewees felt a sense of responsibility to do right by their trial participants. This responsibility seemed to feed into every aspect of the intervention with tremendous thought and effort going into the design, delivery and aftercare of patients, in some cases even once the trial had ended. Interviewees expressed that time commitment was not an issue if a task was deemed to help the trial’s success. Several interviewees advised that future researchers adapt when needed, be patient and prepare for *“bumps in the road”. (P3)*

It was implied that the emotional link and the added sense of responsibility could be difficult for novice researchers, particularly if they do not have experience working with cancer patients, have not received appropriate training or do not have a healthcare background. Debriefings were used by one research team if a researcher felt particularly overwhelmed by a conversation or situation.

*“It’s tough, you know, as you build a relationship with people and they’re maybe confiding in you and issues that are important to them, so yeah, it’s not something anyone can just do….I often said to consultants, you know, there needs to be some training for people who are exposed to this, to this cohort, especially in a kind of close proximity setting that we have in the hospital or in the community…but there’s not.” P7*.

### Study participants

All interviewees felt the social and peer support aspect of an exercise class was as important to a male cohort as to a female cohort.

*“The camaraderie that happens in the class is actually, we have found that it’s almost stronger among the men than the women.” P8*.

Some interviewees found the additional support offered by the research team was often the main draw to signing up rather than the exercise element itself.

*“I found the patients that have said yes, are the kind of patients who do either want or perhaps need a little bit more support.” P2*.

Furthermore, it has been suggested that participants in exercise trials may experience improved healthcare as an indirect benefit, because of receiving additional assessments and increased contact time with research staff. As a result of building close relationships with programme facilitators, participants often disclose personal health concerns that might have gone undetected during routine medical appointments. While interviewees did not observe a gender-based difference in the facilitator’s rapport-building capacity, they suggested that exploring this topic in future research could be intriguing.

## Discussion

This study aimed to explore the experiences of research personnel who have worked with prostate cancer cohorts in exercise oncology research and seek their views on the effective design of trials and exercise interventions.

The findings highlighted recruitment strategies that aided the recruitment of prostate cancer patients to exercise trials. Recruiting directly from hospital clinics and state cancer registries were stated as the most successful and commonly utilised. Embedding the research coordinator into the hospital setting and involving medical personnel in the research team to help build rapport and a sense of ownership in the trial’s success was deemed vital. The importance of sharing recruitment experiences and lessons learnt was similarly highlighted in a trial comparing seven different recruitment strategies targeted at cancer patients and caregivers [25]. Recruitment has been a long-cited problem in the published literature when running clinical trials, leading to methodological limitations in some cases [1]. Common reasons for declining to participate given by prostate cancer patients are similar to those given by other cancer types, which include time and travel burden [26, 27].

Home-based programmes which have become a more common feature in the literature offer a way of combating recruitment barriers [28]. The challenges the interviewees in this study experienced when conducting interventions in a home setting were similar to those highlighted in a recent systematic review that looked at home-based aerobic and resistance exercise interventions in cancer patients and survivors [29]. However, with the advancements in remote monitoring due to the Covid-19 pandemic, these challenges may be more readily overcome. Further research is required to investigate the comparative efficacy of home-based interventions versus supervised trials. Additionally, there is a need for research to determine the setting that results in more sustainable and long-lasting lifestyle changes for participants.

Interviewees working with this cohort identified health issues such as cardiovascular disease, immobility, and arthritis as the primary reasons for deviating from the planned exercise prescription, rather than cancer-related issues. A cautionary note was expressed to future researchers regarding the potential elevated risk of cardiovascular incidents associated with high-intensity exercise prescriptions. Additionally, practical considerations such as the requirement for one-to-one supervision were highlighted. This may be particularly important if targeting prostate cancer patients receiving androgen deprivation therapy (ADT) and radiation therapy as it has been reported that men opting for this treatment option tend to be older, have poorer performance status and unfavourable disease characteristics [30]. ADT has also been reported to increase the risk of coronary heart disease, sudden death and myocardial infarction [31]. Future trials may want to consider, as suggested by some interviewees, incorporating an introductory period to allow the exercise participants to build their confidence, mobility and exercise skillset before attempting to employ desired intensities and volumes.

There is considerable heterogeneity pertaining to exercise outcome measurement tools within published literature [7–12]. This is further corroborated by the findings of this study, which reveal that interviewees base their test selection on factors such as time constraints, professional proficiency, practicality and resource availability. Cancer stage and population age may be an important factor to consider when choosing outcome measurement tests. If targeting a lower risk prostate cancer group and therefore a potentially a younger and fitter cohort, tests of maximal aerobic fitness may be justified. This could potentially lead to standardised outcome tests based on the cancer stage being recruited. While QOL questionnaires were seen as burdensome, interviewees deemed them a vital outcome to measure as prostate cancer patients are at a high risk of psychological distress and poorer quality of life [32, 33].

This study also highlighted the emotional dimensions of working closely with prostate cancer patients in an exercise oncology research setting. The ability to identify and manage emotional and physical issues raised by participants is a critical skill when working with this cohort of patients, and researchers must be prepared to take on this role. However, becoming a support system for their participants can place an emotional strain on the researcher. It has been shown that individuals who lack appropriate training or confidence when managing the emotional demands of patients can experience heightened stress levels and that emotional strain can have a negative impact on healthcare professionals’ health and the quality of care they give [34]. Strategies need to be implemented, such as debriefings and appropriate training to those working in exercise oncology, particularly if they have no prior experience working in a cancer setting.

The findings indicate that social and peer support for patients was perceived as a key benefit to participating in an exercise trial, corroborating previous research [35]. The phenomenon of individuals deriving benefits from shared experiences and supporting others undergoing a comparable cancer journey is well-documented. Exercise interventions could offer a supportive care structure that maybe more appealing to a male cohort compared to a traditional cancer support group [36], and therefore future exercise trials should integrate a social element both for in-person and homebased trials.

This study revealed that increased assessments and interaction with research or healthcare personnel during exercise trials could possibly lead to improved healthcare outcomes. Additional healthcare monitoring, the potential to build a close rapport with the research team and reassurance over health-related issues were all stated as motivators to participation in clinical trials [37].

### Strengths and limitations

This novel qualitative study investigated the practical lessons learnt by researchers carrying out exercise oncology trials with prostate cancer patients. These insights are often not published in research papers but are beneficial to know prior to commencing a similar study with the same cohort. The one-to-one interview structure afforded interviewees an opportunity to voice their own opinion and their own personal experiences.

Limitations of this study included the use of purposive sampling, possibly increasing the risk of selection bias. This study may also have appealed only to individuals willing to share their experiences and motivated to share their advice and therefore may not represent the research community’s views. However, an attempt was made to recruit individuals from different countries to broaden and enrich the data captured.

## Conclusion

Conducting research in exercise oncology and prostate cancer presents significant challenges. Gaining insights from experienced professionals in this field can provide valuable information and guidance on critical aspects such as recruitment, design, health considerations, outcome measures, as well as facilitators and barriers that can influence the success of trials involving men with prostate cancer.

### Electronic supplementary material

Below is the link to the electronic supplementary material.

Supplementary Material 1

## Data Availability

The datasets generated and/or analysed during the current study are not publicly available due to the sensitive nature of the subject matter, which could potentially lead to the identification of the participants but are available from the corresponding author on reasonable request.

## Abbreviations

SRQR: Standards for reporting qualitative research
WIT: Waterford Institute of Technology
DOMS: Delayed onset muscle soreness
QOL: Quality of life
CPET: Cardiopulmonary exercise testing
DEXA: Dual energy x-ray absorptiometry
ADT: Androgen deprivation therapy
