# Supplementary material for: Designing effective exercise intervention trials for prostate cancer cohorts: a qualitative study on experiences and views of exercise oncology researchers
Source: BMC Sports Sci Med Rehabil. 2023 Oct 30;15:145. doi: 10.1186/s13102-023-00756-7 (PMC10614306; doi:10.1186/s13102-023-00756-7)
Supplement: Supplementary file 1 — Supplementary Material 1 [file 13102_2023_756_MOESM1_ESM.docx]

**Additional file 1: Topic guide for interviews**

| **Topic** | **Possible Questions** | **Probes/Prompts** |
| --- | --- | --- |
| Demographic | 1. *Tell me a bit about yourself?* 2. *What are your thoughts on the qualifications and experience that is needed to conduct exercise trials on prostate cancer?* | *Primary role in the study.*  *Qualifications or any specific exercise oncology training?* |
| Recruitment | 1. *Tell me about your experiences with recruitment. What works, what doesn’t work?* 2. *What do you think is the optimal way to recruit patients?* | *What are the typical reasons why patients do not want to participate in research?* |
| Retention and behavioural change/support | 1. *Tell me about your approach to motivating and retaining participants.* 2. *What are the primary reasons for dropout?* | *Group verses individual training*  *Social element:*  *important to men?*  *Behavioural elements: important?*    *Mixed sex groups vs males only*  *Mixed cancer groups vs prostate cancer only?* |
| Program Goals | 1. *What do you believe should be the primary goal of exercise interventions for prostate cancer patients?* | *Aerobic vs resistance exercise?*  *If a mix of modalities, how do you mix them (i.e., split session, or dedicated day? etc)*  *Enjoyment vs maximum exercise adaptation* |
| Research Design | 1. *What are your thoughts on stratifying and randomising?* 2. *What in your opinion is the most ideal time to start an exercise intervention for prostate patients (e.g., start of androgen deprivation therapy (ADT), post Radiation therapy RT)?* | *Mid ex assessment if patients have block of RT during intervention*  *Did you exclude if on active treatment or exclude if cycling off ADT before final assessment?*  *Would you change or do anything differently?* |
| Special/Disease Considerations | 1. *What are the specific issues that pertain to prostate patients (with regards their disease) in your experience that impact exercise?* 2. *How did you deal with these specific issues?* 3. *Do you have any experience with patients with bone metastases?* | *Bone metastases: specific exercise considerations, how were they accommodated?*  *Pelvic floor exercises?*  *Incontinence issues?* |
| Treatment Considerations | 1. *Have you any experience with patients on ADT and if so any special considerations with regards exercise prescription?* 2. *Did any of your participants have active treatment during the intervention such as chemotherapy or radiation therapy? If so, did you have to adapt your exercise prescription because of this?* | *Radiotherapy given during intervention (Special considerations: patients on special diet, can’t lose weight due to impact on treatment, hydration levels, increase fatigue, time constrains)?*  *Types of ADT, any specific considerations between different types of ADT?* |
| Adverse Events | 1. *Are there specific adverse events that we need to be mindful of when working with prostate patients* 2. *Is there anything you feel you put in place that help to reduce the risk?* | Any checks that you do at the start of a session?    Under what circumstances might you refer back to the patient’s medical team? |
| Exercise Prescription | 1. *What approach works best in terms of FITT and in terms of starting dose and progression* 2. *How did you monitor adherence (attendance vs % of ex prescription completed)?* 3. *Have you any experience with home-based programmes? Can they work?* | *How did you determine the initial load (For both aerobic and resistance)?*  *Why did you feel that this was the most appropriate strategy?*  *Structuring of session, introducing variety*  *Free vs machine weights vs bands*  *Use of buddies, little bit of competition, i.e., behaviour change strategies)*    *What do you think are the strengths and limitations of home-based programs for this population?* |
| Outcome tools | 1. *What do you believe are the best measurement tools to use in terms of:*   *Cardiovascular fitness*  *Strength*  *Physiological function, Fatigue /quality of life, Body comp*  *Blood biomarkers?* | *Any benefit in measuring prostate biomarkers?*  *Questionnaire based assessments.*  *Sexual function?*  *Issues and any strategies you found helped?*    *Bike verses treadmill cardiovascular fitness tests* |
| Final questions | 1. *For someone who is about to embark on an exercise oncology study for prostate cancer patients, have you any additional advice you would like to give them?* 2. *If you could change anything in your own study, what might that be?* 3. *What are the big research questions that remain to be answered in relation to Exercise and prostate cancer* |  |
